# Analysis of the Sense of Occupational Stress and Burnout Syndrome among Working Physiotherapists—A Pilot Study

**DOI:** 10.3390/medicina57121290

**Published:** 2021-11-24

**Authors:** Joanna Kowalska, Daniel Chybowski, Dorota Wójtowicz

**Affiliations:** Department of Physiotherapy, University of Health and Sport Sciences, 51-612 Wroclaw, Poland; joanna.kowalska@awf.wroc.pl (J.K.); dan.chybowski@gmail.com (D.C.)

**Keywords:** burnout syndrome, coping strategies, Perceived Stress, physiotherapist, rehabilitation, work characteristics, work stress

## Abstract

*Background and Objectives*: As medical service employees, physiotherapists are prone to suffer from job-related stress and are at great risk of experiencing occupational burnout. Therefore, the aim of this pilot study was to evaluate the level of generalized stress, occupational burnout syndrome and occupational stress in a group of professionally active physiotherapists and to answer the question: which psychosocial and physical factors (work characteristics) present at the given workplace were perceived as the most stress-inducing in the study group and in various subgroups? *Materials and Methods*: This study included 70 physiotherapists, mean age 40.1 ± 11.6, employed in sanatoria and outpatient clinics. An authorial survey, the Subjective Assessment Work Questionnaire, the Oldenburg Burnout Inventory, the Perceived Stress Scale, and the Inventory to Measure Coping Strategies with Stress Mini-COPE were used. *Results*: The study group of physiotherapists was characterized by a moderate level of stress, a high level of occupational stress and a moderate level of occupational burnout. The most common stressors reported by the participants included the lack of rewards at work, the sense of uncertainty resulting from workplace organization, the sense of threat, social interaction, and the lack of control. *Conclusions*: The knowledge of the level of occupational stress among health care professionals (including physiotherapists) and, most importantly, the assessment of stress-inducing psychosocial and physical factors present at the given workplace may prove useful while designing a prevention and health protection strategy.

## 1. Introduction

Occupational burnout is a syndrome characterised by emotional exhaustion, depersonalisation and a reduced sense of personal accomplishment. Emotional exhaustion caused by stressful work is characterised by fatigue, reduced motivation to act, irritability, lack of vigour, and diverse psychosomatic symptoms. Due to the development of defensive mechanisms as protection against the consequences of exhaustion, patients experience depersonalisation manifested as indifference and distancing from other people—often those who use their services. Reduced sense of personal accomplishment is characterised by the employee’s decreased performance and underestimation of their own skills [1,2].

Furthermore, occupational burnout syndrome has not been clearly defined yet. The existing review papers contain over 100 various definitions, as well as describing diverse methods for evaluating occupational burnout [3].

Professionals whose work involves helping others are particularly vulnerable to occupational burnout syndrome [4]. One of the high-risk groups are health care professionals [5]. Medical staff are exposed to occupational and emotional stress, caused chiefly by their ongoing contact with patients who expect help and care. They experience both a sense of responsibility for their patients’ life and health and a sense of helplessness when the demands and expectations of the patients and their loved ones cannot be met by modern medicine. This gives rise to “defensive dehumanisation” that serves as protection from excessive emotions by way of perceiving the patients as objects undergoing medical procedures rather than as human beings. As a consequence, health care professionals talk about “cases” or “symptoms” and not about suffering individuals [1,6]. This is also observed in physiotherapists who are members of therapeutic teams. 

According to Rasmus et al., occupational burnout and a high level of stress also affects young people, in particular medical students, including physiotherapy students [7,8,9]. 

In order for a physiotherapist to properly perform their professional duties, they need to be fully involved in the relationship with the patient and their family. Improper working conditions, lack of time, occupational overload and burnout experienced by a physiotherapist can have an adverse impact not only on the effectiveness of the therapy, but also (in a long-term perspective) on the independence and quality of life of the patient and the physiotherapist alike [10].

Researchers confirm the existence of occupational burnout and a high risk of its occurrence [2,10,11,12,13,14,15]. The studies carried out by Carpi et al. and Durand et al. demonstrated that 14% and 19.3% of the employees, respectively, were exposed to a high risk of occupational burnout [16,17]. Baudry et al. found a high risk of burnout in 25% of participants [18]. In the studies carried out by Deneva et al., in terms of emotional exhaustion, 60.7% of physicians showed a high score [19].

Pustułka-Piwnik et al. demonstrated in their studies that the indicators of occupational burnout in physiotherapists are significantly correlated with selected demographic and organisational variables. Occupational burnout manifested as a higher level of emotional exhaustion and reduced satisfaction in accomplishments [2]. Mikołajewska emphasises that the nature of physiotherapists’ work increases the risk of occupational stress and burnout. Due to occupational stress and burnout, 11–16% of Polish physiotherapists (5500–8000 individuals) make a career change [10].

There is a correlation between occupational burnout and the level of stress and the emerging occupational stress. In response to the growing number of employees diagnosed with stress-induced conditions, the interest in the issue of occupational stress has also been increasing [20,21]. This results from the ever-changing tasks and working conditions, brought about e.g., by privatisation, automation and globalisation [22].

The correlation between the exposure to occupational stress and the level of burnout among health care professionals was confirmed in the research conducted by Carmona-Barrientos et al. [14]. However, it was not confirmed by Boland et al. [23].

Therefore, it is necessary to conduct further research on mental and physical health of professionals working in medical institutions [12]. The knowledge of the level of occupational stress among health care professionals and, most importantly, the assessment of stress-inducing psychosocial and physical factors present at the given workplace may prove useful while designing a prevention and health protection strategy. 

The issue of stress and occupational burnout and their correlation with the level of perceived occupational stress among physiotherapists is rarely discussed in the literature. Therefore, the aim of this pilot study was to evaluate the level of generalised stress, occupational burnout syndrome and occupational stress in a group of professionally active physiotherapists and in various subgroups based on gender, place of employment, age and years of professional experience and to answer the following questions: 

What was the general level of burnout syndrome and occupational stress, and which work characteristics were perceived as the most stress-inducing in the sample group of physiotherapists?

What strategies for coping with stressful situations were employed by the participating physiotherapists?

What is the relationship between occupational burnout syndrome and generalised stress, as well as the subjective level of perceived occupational stress in the sample group of physiotherapists? 

Which factors (work characteristics) present at the given workplace are associated with occupational burnout in the sample group of physiotherapists? 

## 2. Materials and Methods

### 2.1. Participants

The pilot study was conducted in the Lower Silesian Voivodeship between November 2019 and February 2020. The respondents were selected based on the non-random snowball sampling model, in which participants recruit other participants for the study. 

The study group consisted of 70 professionally active physiotherapists, including 56 female and 14 male participants. The mean age was 40.1 (11.6) years. Nearly 73% of the participants were employed in sanatoriums and 27% in outpatient clinics. Sixteen participants worked in their profession for up to 5 years; 18 participants—between 6 and 10 years; 8 participants—between 11 and 15 years; 6 participants—between 16 and 20 years; and 22 participants—over 21 years (Table 1).

### 2.2. Procedure

The research procedures were compliant with the standards of the Senate Commission for the Ethics of Scientific Research of the University School of Physical Education in Wrocław and with the Declaration of Helsinki [24]. The study was conducted in the form of anonymous questionnaires, without any intervention or experiment, with the consent of all participants and an option to withdraw from the study without providing a reason and without any adverse consequences. It was carried out under the supervision of the Department of Physiotherapy of the University School of Physical Education in Wrocław and did not require ethical assessment.

### 2.3. Measurement Tools

The following tools were used in the study: the Subjective Work Assessment Questionnaire (SWAQ), the Oldenburg Burnout Inventory (OLBI), the Perceived Stress Scale (PSS-10) and the Brief Coping Orientation to Problem Experienced (Mini-COPE) inventory. Sociodemographic data and information concerning the participants’ work were collected using the authors’ own questionnaire composed of 15 closed-ended questions.

The SWAQ, created by Dudek et al., is used to subjectively assess the onerousness of work and measure the individual perception of stress at the workplace. It examines generalised stress and identifies the most stress-inducing psychosocial and physical factors present at the workplace. It consists of 57 questions that address various characteristics of work, classified into 10 factors: sense of mental strain associated with the complexity of work, lack of rewards at work, sense of uncertainty resulting from workplace organisation, social interactions, sense of hazard, physical inconvenience, unpleasant working conditions, lack of control, lack of support, and the sense of responsibility. Respondents answered the questions using a scale from 1 to 5 to reflect the level of onerousness of the given characteristic, where 1 represents the absence of the given factor and 5 represents the highest level of irritation with the given characteristic and its very high onerousness. The total number of points indicates the level of occupational stress experienced by the respondent. The higher the score, the more intense the stress. Furthermore, the total number of points for individual factors, compared with the values included in the standards table compiled by the authors of the questionnaire, indicates the groups of factors that pose a particular hazard to the employee. Cronbach’s alpha for the questionnaire as a whole is 0.84, and for individual factors, it ranges between 0.49 and 0.83 [22]. 

The OLBI created by Demerouti is used to evaluate the level of occupational burnout in professionally active individuals. It comprises two subscales—exhaustion and disengagement (cynicism). Exhaustion refers both to physical fatigue, strain and lack of energy, and to mental and emotional overload and exhaustion associated with work, both physical and intellectual. Disengagement is the consequence of long-term stress, i.e., mental and physical tension, where the individual withdraws and distances themselves from their work, exhibits cynicism, experiences dehumanisation in social interactions at work, and loses their ideals and the intention to continue their career [25]. The tool consists of 16 questions, 8 for each subscale. The scores are calculated for each subscale. The higher the score, the higher the level of occupational burnout. In the Polish adaptation by Chirkowska-Smolak, Cronbach’s alpha was over 0.7 for the scale as a whole, 0.75 for the exhaustion subscale and 0.74 for the disengagement subscale [26].

The PSS-10, created by Cohen et al., consists of 10 questions and measures the subjective perception and feelings associated with stressful situations, as well as the coping mechanisms. The higher the score (max. 40 points), the higher the intensity of experienced stress. The raw score is interpreted using a sten scale where a sten score of 1–4 (0–13 points) is considered low, a sten score of 5–6 (14–19 points) is considered moderate, and a sten score of 7–10 (20–40 points) is considered high. The authors of the Polish adaptation of the test are Juczyński and Ogińska-Bulik; Cronbach’s alpha was 0.86 [20].

The Mini-COPE inventory created by Carver is used to evaluate typical coping strategies and reactions in situations where the individual experiences severe stress. It consists of 28 statements that refer to 14 strategies describing various coping styles. The higher the score, the more frequently the given strategy is employed by the respondent. Strategies such as active coping, planning and use of instrumental support are referred to as “problem-focused”. Use of emotional support, religion, or denial are considered “emotion-focused”. Venting, self-distraction, behavioural disengagement, substance use and the sense of humour signify avoidance; nevertheless, they can bring short-term relief. Split-half reliability was 0.86 (Guttman split-half coefficient—0.87) [20].

The questionnaire compiled by the authors was composed of 15 closed-ended questions—in addition to the questions on basic personal details—and concerned workplace-related issues, such as the choice of current workplace, superiors’ engagement in their staff’s professional development, remuneration, difficulty of work duties, feeling appreciated by the employer, nature of the work, use of the employee’s competence, knowledge and skills, workplace organisation and work schedule, and the perception of time pressure that might affect work quality.

### 2.4. Statistical Analysis

The statistical analysis was carried out using descriptive statistics such as mean, standard deviation and—in the case of qualitative variables—percentages and amounts. Normality of distribution was verified using the Kolmogorov–Smirnov test. Due to the small size of subgroups, non-parametric tests were used during the statistical analysis despite normal distribution. The results in two groups of physiotherapists were compared using the Mann–Whitney U test and the Kruskal–Wallis test for more than 2 groups. The strength of correlation between selected pairs of variables was assessed using Spearman correlations. Statistical tests were verified at the significance level of *p* < 0.05.

## 3. Results

The statistical analysis included the responses of 70 professionally active physiotherapists. A description of the study group of physiotherapists is detailed in Table 1.

Taking into consideration the data collected through the questionnaire, it was concluded that the primary motivations behind seeking and obtaining employment were: proximity to the place of residence (38.6%), personal interests (35.7%), and lack of other job opportunities (17.1%). The majority of the respondents (62.9%) had a negative opinion on their superiors’ engagement in the employees’ professional development. The overwhelming majority was dissatisfied with their remuneration and felt underappreciated by their employers (67.1%). As many as 75.7% of the respondents defined their work as imitative and consisting in the performance of precisely communicated procedures. Another concerning finding is that a high percentage of physiotherapists (65.7%) felt they were under time pressure at work, which may have a negative impact on the quality of the services they provide to the patients. Detailed results are presented in Table 2.

The mean level of stress (PSS-10) among the physiotherapists was 18.0 (6.5). The largest number of individuals (*n* = 28; 40%) was characterised by a high level of stress. Nearly 40% (*n* = 22) of female respondents were experiencing a high level of stress at the time of the study. There were no statistically significant differences in the level of stress between the groups of female and male respondents (Table 3).

The mean level of occupational burnout (OLBI) in the exhaustion subscale was 2.6 (0.4) and in the disengagement subscale—2.5 (0.4). The differences between the groups of female and male respondents in both subscales were not statistically significant. Physiotherapists working in sanatoriums were characterised by a higher level of occupational burnout than the specialists working in outpatient clinics. In particular, statistically significant differences were found in the disengagement subscale of OLBI (Table 3).

The differences in the level of occupational burnout between the groups of respondents based on the age and years of professional experience were not statistically significant (OLBI—exhaustion: H = 2.8; *p* = 0.2390; disengagement: H = 0.4; *p* = 0.8181; OLBI—exhaustion: H = 4.8; *p* = 0.0905; disengagement: H = 0.2; *p* = 0.9036, respectively).

The mean value of occupational stress (SWAQ) in the sample group of physiotherapists was 125.4 (33.7), i.e., a sten score of 7.6, which corresponds to a high level of stress. The differences between the groups based on gender were not statistically significant (z = 0.02; *p* = 0.8723). Furthermore, there were no statistically significant differences in the level of occupational stress between the groups based on age, years of professional experience and the place of employment (H = 0.6; *p* = 0.7508; H = 0.3; *p* = 0.8443; z =0.5; *p* = 0.4688, respectively).

Highly stress-inducing factors in the sample group of physiotherapists included: lack of rewards at work, sense of uncertainty resulting from workplace organisation, social interactions, sense of hazard, lack of control, lack of support, and the sense of responsibility.

The group of physiotherapists working in sanatoriums significantly more frequently pointed to the following stress-inducing work characteristics: physical inconvenience, unpleasant working conditions and lack of control, as compared to the physiotherapists working at outpatient clinics. Detailed data are presented in Table 4.

The most common strategies used by the sample group of physiotherapists in situations that generate severe stress included active coping, planning, use of emotional support, positive reframing, and acceptance. The comparison of the groups of female and male participants revealed a statistically significant difference in coping strategies such as the use of emotional support, the use of instrumental support, and self-distraction.

In physiotherapists working at outpatient clinics, the most frequently applied strategies included active coping, acceptance, and planning. Physiotherapists working at sanatoriums used the following techniques: active coping, planning, and the use of emotional support.

The comparison of the groups of physiotherapists based on age and years of professional experience revealed a statistically significant difference in the venting strategy. Detailed data are presented in Table 5.

Correlation analysis demonstrated a statistically significant positive correlation between SWAQ scores and PSS-10 scores, and between SWAQ scores (and their factors) and OLBI scores in both the exhaustion and disengagement subscales, as well as a statistically significant positive correlation between PSS-10 scores and OLBI scores in the exhaustion subscale (Table 6).

## 4. Discussion

The results of the pilot study demonstrated that physiotherapists belong to the group of occupations at increased risk associated with the presence of psychosocial hazards at work. This is evidenced by a high level of stress experienced by the physiotherapists participating in the study, and the intensity of workplace stressors. The respondents were also characterised by a high level of occupational stress (SWAQ score). Conversely, PSS-10 scores indicated a moderate level of generalised stress; however, those values bordered on the high level. Furthermore, correlation analysis demonstrated that the greater the employee’s exposure to psychosocial hazards at the workplace, the greater their sense of generalised stress and the higher their level of occupational burnout. If that situation persists long enough and no appropriate preventive measures are implemented, the employees may experience somatic, mental and social symptoms of chronic stress and, consequently, develop the occupational burnout syndrome [27]. In turn, occupational burnout may exacerbate health, family, and social problems [28]. A positive correlation between the PSS-10 score and the intensity of occupational stress (SWAQ score) was also observed by Juczyński and Ogińska-Bulik [20]. A high level of occupational stress among physiotherapists working at hospital departments was also confirmed by Humeniuk et al. [21]. Research conducted by Sochacka et al. revealed that as many as 99% of the respondents reported the presence of stress at work, and 62% of the respondents described the level of stress as high. This demonstrates that the profession of physiotherapist is one of the occupations that are characterised by an increased risk of occupational stress [29]. Conversely, research conducted in Spain on 272 physiotherapists working in the public and private sectors showed that 30.51% of physiotherapists experienced a high level of occupational stress and 34.56%—a moderate level of occupational stress. The overall level of occupational burnout was low because the only high value was that of emotional exhaustion, while the values of depersonalisation and the reduced sense of personal accomplishment were low. A correlation between occupational stress and occupational burnout was demonstrated; this confirms that the cumulative effect of stress may lead to burnout [14].

Among the physiotherapists who participated in the study, the most popular stress-coping strategies were active coping, planning, use of emotional support, acceptance, and positive reframing. Active strategies, such as active coping, planning or positive reframing are generally associated with a lower level of stress. Conversely, the use of emotional support, also reported by the participants, is an emotion-focused activity that may prove to be an adaptive form of coping, although this is not obvious [20]. In practice, this means that physiotherapists should have an opportunity to receive psychological help or join support groups. The results of Carmona-Barrientos et al., regarding occupational stress, show the necessity of developing coping strategies for physiotherapists and health care professionals [14]. Unfortunately, research shows that only a fraction of the participants (approximately 15%) seeks and receives psychological support by seeing a psychologist or a therapy group in their struggle with stress [21,29].

The study revealed that female participants coped with difficult situations in a different way than male participants. Female physiotherapists significantly more frequently sought emotional and instrumental support and used the self-distraction strategy, as compared to male physiotherapists. Such substantial differences were not recorded between groups of physiotherapists based on age, place of employment or years of professional experience. The only observation in this regard was that the youngest group of physiotherapists and the group with the shortest professional experience significantly more frequently employed the venting strategy as compared to older and more experienced physiotherapists.

Positive reframing was recorded in the oldest respondents (aged 41–63) and the respondents with the longest professional experience (over 20 years). This is likely the result of the rich and diverse life, personal, and professional experience of those respondents. A positive surprise is the fact that health-damaging practices, such as substance use, behavioural disengagement, denial, and self-blame were followed quite rarely by the participants. Only the physiotherapists working at outpatient clinics significantly more frequently employed the substance use strategy as compared to the physiotherapists working in sanatoriums. This is concerning but most likely requires further verification using a larger group of physiotherapists.

The participants demonstrated a moderate level of occupational burnout in both the disengagement scale and the exhaustion scale. Nonetheless, those scored bordered on high values. Both female and male physiotherapists achieved similar scores in terms of occupational burnout. However, in female respondents social interaction appeared to be more stress-inducing than in men (SWAQ score), which may suggest that their disengagement is a mechanism of defence against stress experienced during social interaction at work. Furthermore, such defence mechanisms play an important adaptive role and protect self-esteem [30]. In the study carried out by Lee et al., female therapists showed higher levels of burnout than male therapists [15]. Conversely, Bejer et al. demonstrated that professional burnout is most likely to occur in men [31]. The analysis of occupational burnout scores in individual age groups did not reveal any significant differences, contrary to the research conducted by Wrzesińska et al., who observed that the level of burnout increases with age [32].

The length of professional experience also did not affect the level of occupational burnout among the participating physiotherapists; in contrast, the studies carried out by Bejer et al. demonstrated that longer work experience was associated with a higher risk of occupational burnout [31]. In the results of the study carried out by Pniak et al., the highest level of burnout was also manifested by the physiotherapists who had been professionally active for more than 20 years [33]. As mentioned before, the positive reframing strategy employed by the oldest and the most experienced physiotherapists (over 21 years of professional experience) is a unique characteristic of emotionally mature individuals. Stanton and Snider demonstrated that the positive reframing strategy is associated with less intense stress, which, in turn, leads to a lower level of occupational burnout [34]. Li et al. emphasised that positive coping strategies reduce or buffer the negative effects of occupational stress and negative coping strategies increase the negative effects [35].

Physiotherapists working at sanatoriums were characterised by a significantly higher level of burnout in terms of disengagement than the physiotherapists working in outpatient clinics. In terms of exhaustion, the level of burnout was similar. Physiotherapists working in sanatoriums reported nearly twice as often as the physiotherapists working in outpatient clinics that their work is imitative and that they work under time pressure that may have a negative impact on the quality of the services they provide. Furthermore, physiotherapists working in sanatoriums significantly more frequently reported workplace stressors such as physical inconvenience, unpleasant working conditions, and lack of control. Sanatoriums are unique workplaces where physiotherapists are exposed to high humidity and slippery surfaces (resulting in the risk of falling). Those facilities are also characterised by a specific microclimate resulting from the presence of gases such as hydrogen sulphide, radon or carbon dioxide. Furthermore, the physiotherapist’s control over the rehabilitation process in a sanatorium might be severely restricted. Rehabilitation procedures are precisely ordered by physicians and there is rarely any direct feedback on the effectiveness of treatment, because the results usually appear after some time, when the patient is already back home [36].

Pustułka-Piwnik et al. found that physiotherapists who work with adult patients and are employed at hospitals experience a higher level of emotional exhaustion and depersonalisation. Depersonalisation was also observed in individuals with 15–19 years of professional experience. A reduced sense of personal accomplishment was confirmed in physiotherapists with a lower level of education [2]. Physiotherapists working in the private sector in France were found to be at a greater risk of the occupational burnout syndrome as compared to the physiotherapists working in the public sector [18].

A similar level of exhaustion was observed in the group of hospital nurses [37]. Similarly, the scores related to disengagement were generally higher than in other categories of professionals, such as physicians, teachers, kindergarten teachers or therapists working with children suffering from autism [38]. A higher level of burnout as compared to the respondents participating in the authors’ study was observed in the physiotherapists from the Łódź Voivodeship in the study conducted by Wrzesińska et al. [32]. Conversely, the participants of the study conducted by Kowalska (*n* = 64) achieved relatively low occupational burnout scores. The reason behind the better condition of those physiotherapists was higher job satisfaction and better working conditions in facilities located in Warsaw as compared to smaller health care centres [11].

The lack of rewards at work is the most stress-inducing factor at work, irrespective of the respondents’ gender, age, place of employment and years of professional experience. To emphasise the negative and demotivating impact of this factor it should be noted that a vast majority of respondents (over 60%) viewed their remuneration as low or very low, felt underappreciated by their employer and had a negative opinion on their employers’ engagement in the staff’s professional development. Furthermore, the correlation analysis performed showed a strong association between this characteristic and the occurrence of occupational burnout in the participants.

Long-term exposure of the employees to highly stress-inducing factors at work may have a profound impact on the development of the occupational burnout syndrome in physiotherapists. This is confirmed by the positive correlation between occupational burnout (OLBI score) and the perceived level of occupational stress (SWAQ score). Similar results were obtained by Chirkowska-Smolak, as well as Baka and Basińska and Carmona-Barrientos et al. [14,26,39].

Findings concerning occupational burnout often vary considerably. This is undoubtedly the result of the diverse functioning of and inconsistency between various elements the health care system in Poland and worldwide, the difference between remuneration levels in the public and private sectors, the unique economic situation in individual countries and the differences between the research methodology adopted by the authors of publications.

Unfortunately, the consequences of occupational burnout affect not only the professionals who experience it, but also their friends, family and the people they meet at work (colleagues and patients). In addition to the risk of development of psychosomatic conditions, those professionals often suffer from depression, marital and family problems, as well as social alienation and the desire to make a career change [10]. The economic burden of occupational burnout is carried by the entire society. The main sources of financial losses are the decreased effectiveness and performance, as well as staffing problems and, in some cases, the costs of retirement and disability pensions. The absence of employees caused by a large number of sick leaves and a long treatment process require the employers to seek and hire new staff. Therefore, further research on the prevention of occupational stress and burnout among physiotherapists is economically and socially justified.

A modern strategy for protecting the employees’ health should be based on monitoring and minimising all occupational hazards, including psychosocial and physical factors at the workplace. Occupational stress prevention programmes should be based on the systemic identification of hazard factors, planning and design of intervention methods, implementation of those solutions and their evaluation and control in order to verify their effectiveness.

The study discussed in this article had certain limitations. First and foremost, it was a pilot and screening study, and the sample group of physiotherapists was diverse and quite small. Therefore, the sample group cannot be considered as representative. The findings should be construed as preliminary, and they should not be generalised. Nevertheless, they already justify further research into this issue. Based on these findings, the authors intend to increase the size of the sample group and include physiotherapists working at other medical facilities in the Lower Silesian Voivodeship in order to use more advanced statistical analyses. The researchers who study the occupational burnout syndrome and occupational stress employ various research methodologies, which hinders the comparison of the findings.

## 5. Conclusions

The study group of physiotherapists was characterised by a moderate level of stress, as measured using PSS-10, a high level of occupational stress (SWAQ score) and a moderate level of occupational burnout.

The most common stressors reported by the participants included the lack of rewards at work, the sense of uncertainty resulting from workplace organisation, the sense of threat, social interaction, and the lack of control.

Physiotherapists working in sanatoriums significantly more frequently than those working at outpatient clinics described factors such as physical inconvenience, unpleasant working conditions and the lack of control as highly stress induing.

In the study group, a higher level of generalised stress and a high level of occupational stress correlated with a higher level of occupational burnout.

These findings pertain only to the study group and should be confirmed or disproved in research on a larger group of physiotherapists.

## Figures and Tables

**Table 1:** Characteristics of the study group (N = 70).

Feature	M	SD
Age	40.1	11.6
Years of work experience	13.0	8.0
Workplace	*n*	%
sanatorium	51	73
private outpatient clinic	12	17
public outpatient clinic	7	10
Marital status		
single	15	21
married	46	66
divorced	9	13
widowed	0	0
Education		
physiotherapy technician	19	27
BSc in physiotherapy	23	33
MSc in physiotherapy	28	40
Additional work		
Yes	19	27
No	51	73
Work on Saturdays and Sundays		
Yes	36	51
No	13	19
Occasionally	21	30

**Table 2:** Results of the authors’ own questionnaire.

Feature	*n*	%
Reasons for choosing current place of employment?		
personal interests	25	35.7
remuneration	0	0.0
high quality of provided services	3	4.3
proximity to place of residence	27	38.6
lack of other employment opportunities	12	17.1
other	3	4.3
Opinion on supervisors’ engagement in employees’ professional development		
positive	26	37.1
negative	44	62.9
Opinion on remuneration		
high	1	1.4
adequate	17	24.3
low	32	45.7
very low	20	28.6
Assessment of the level of difficulty of work duties		
adequate for employee’s level of competence	51	72.9
below employee’s level of competence	18	25.7
above employee’s level of competence	1	1.4
Feeling of being appreciated by the employer		
yes	23	32.9
no	47	67.1
Assessment of work character		
creative	17	24.3
imitative	53	75.7
Does the respondent make use of all of their knowledge and skills at work?		
yes	35	50
no	35	50
Opinion on workplace organisation and work schedule		
positive	40	57.1
negative	30	42.9
Does the respondent feel time pressure which could influence the quality of their performance at work?		
yes	46	65.7
no	24	34.3

**Table 3:** The PSS-10 and the OLBI results in the study group of physiotherapists and in selected subgroups (the Mann–Whitney U test).

			Gender						Workplace					
Scales	All Group N = 70		Female *n* = 56		Male *n* = 14				Outpatient Clinic *n* = 19		Sanatorium *n* = 51			
	M	SD	M	SD	M	SD	Z	*p*	M	SD	M	SD	Z	*p*
PSS-10 (Raw score)	18.0	6.5	17.7	6.5	19.1	6.7	−0.4	0.3336	18.9	6.9	17.6	6.4	−0.6	0.2776
OLBI-Exhaustion (Raw score)	2.6	0.4	2.6	0.4	2.6	0.4	−0.5	0.3121	2.6	0.4	2.6	0.4	−0.1	0.4761
OLBI-Disengagement (Raw score)	2.5	0.4	2.6	0.4	2.5	0.6	0.3	0.4013	2.3	0.5	2.7	0.3	3.1	0.0009 **

PSS-10, Perceived Stress Scale; OLBI, Oldenburg Burnout Inventory; ** statistically significant values *p* < 0.05.

**Table 4:** The SWAQ results in the all study group and in selected subgroups (the Mann–Whitney U test).

SWAQ	All Group N = 70		Gender				Workplace						Age						Years of Professional Experience					
			Female *n* = 56		Male *n* = 14		Outpatient Clinic *n* = 19		Sanatorium *n* = 51				20–30 Years Old *n* = 20		31–40 Years Old *n* = 19		41–63 Years Old *n* = 31		5–10 Years *n* = 34		11–20 Years *n* = 14		Over 20 Years *n* = 22	
	M	SD	M	SD	M	SD	M	SD	M	SD	Z	*p*	M	SD	M	SD	M	SD	M	SD	M	SD	M	SD
Total score for occupational stress (raw score)	125.4	33.7	126.2	34.3	122.3	32.1	124.1	38.6	125.9	32.1	0.5	0.3191	121.2	34.5	126.6	31.3	127.3	35.3	127.3	33.4	122.6	30.4	124.2	37.2
Feeling of psychological strain related to work complexity	16.9	6.2	16.8	6.4	17.2	5.5	18.6 *	7.1	16.2	5.7	−1.2	0.1075	15.9	5.7	16.3	6.0	17.8 *	6.6	16.6	5.9	16.1	5.4	17.9 *	7.1
Lack of rewards at work	18.2 *	6.4	18.4 *	6.2	17.4 *	7.3	17.3 *	7.1	18.6 *	6.2	0.7	0.2514	17.5 *	6.0	16.8 *	6.2	19.5 *	6.7	18.5 *	5.6	17.6 *	8.2	18.2 *	6.6
Feeling of uncertainty resulting from workplace organisation	17.3 *	5.7	17.4 *	5.8	16.9 *	5.6	16.5 *	5.5	17.6 *	5.8	0.7	0.2611	16.1 *	5.5	17.7 *	4.8	17.8 *	6.5	17.2 *	5.1	17.7 *	5.8	17.2 *	6.8
Social interactions	9.9 *	3.1	10.0 *	3.3	9.5 *	2.1	10.3 *	3.3	9.8 *	3.1	−0.4	0.3557	9.3 *	3.1	10.6 *	3.7	9.9 *	2.7	10.3 *	3.7	9.4 *	1.7	9.8 *	2.9
Feeling of threat	10.9 *	3.4	10.9 *	3.5	11.0 *	2.9	10.2 *	3.1	11.2 *	3.4	1.1	0.1314	11.4 *	3.2	11.9 *	2.8	9.9	3.6	11.8 *	2.8	10.4 *	3.1	9.9	4.1
Physical inconvenience	6.8	3.2	7.1	3.4	5.7	1.2	5.6	1.9	7.3	3.4	2.0	0.0222 **	6.6	4.2	6.9	3.6	6.9	1.9	6.7	3.4	7.1	4.2	6.9	1.9
Unpleasant work conditions	5.1	2.3	5.2 *	2.4	4.6	1.9	4.1	1.5	5.4 *	2.4	2.3	0.0104 **	5.1 *	3.3	5.2 *	2.1	4.9	1.5	5.3 *	2.8	5.1 *	2.1	4.8	1.5
Lack of control	9.6 *	2.6	9.8 *	2.6	8.7 *	2.6	8.5 *	2.6	9.9 *	2.5	2.2	0.0158 **	9.8 *	3.3	9.5 *	2.7	9.4 *	2.1	9.8 *	2.9	9.2 *	2.8	9.3 *	2.1
Lack of support	5.4 *	2.2	5.4 *	2.1	5.3 *	2.6	5.6 *	2.0	5.3 *	2.3	−0.8	0.2148	5.2 *	2.1	5.5 *	2.3	5.4 *	2.3	5.6 *	2.3	5.2 *	2.3	5.3 *	2.1
Sense of responsibility	8.3 *	2.9	8.3 *	2.9	8.3 *	3.1	7.9	3.4	8.4 *	2.7	0.9	0.1685	7.8	2.5	8.4 *	2.8	8.5 *	3.2	8.3 *	2.6	7.0	3.0	9.1 *	3.1

SWAQ, Subjective Work Assessment Questionnaire; * high stress-inducing factors; ** statistically significant values *p* < 0.05.

**Table 5:** The Mini-COPE results in the all study group and in selected subgroups (the Mann–Whitney U test and the Kruskal–Wallis test).

Mini-COPE	All Group N = 70		Gender						Workplace						Age								Years of Professional Experience							
			Female *n* = 56		Male *n* = 14				Outpatient Clinic *n* = 19		Sanatorium *n* = 51				20–30 Years Old *n* = 20		31–40 Years Old *n* = 19		41–63 Years Old *n* = 31				5–10 Years *n* = 34		11–20 Years *n* = 14		Over 20 Years *n* = 22			
	M	SD	M	SD	M	SD	z	*p*	M	SD	M	SD	z	*p*	M	SD	M	SD	M	SD	H	*p*	M	SD	M	SD	M	SD	H	*p*
1. Active coping	2.1 *	0.5	2.1 *	0.5	2.2 *	0.6	−0.5	0.5961	2.1 *	0.5	2.1 *	0.6	0.4	0.6455	2.1 *	0.7	2.0 *	0.6	2.1 *	0.4	0.5	0.7851	2.1 *	0.7	1.9 *	0.5	2.1 *	0.4	2.1	0.3536
2. Planning	2.1 *	0.5	2.1 *	0.5	1.8 *	0.7	1.4	0.1527	1.9 *	0.6	2.1 *	0.6	1.1	0.2585	2.2 *	0.6	1.9 *	0.7	2.1 *	0.5	2.7	0.2611	2.1 *	0.6	1.7	0.6	2.1 *	0.4	3.3	0.1941
3. Positive reframing	1.8	0.6	1.8	0.6	1.6	0.8	0.9	0.3576	1.8	0.5	1.8	0.7	0.3	0.7565	1.8	0.8	1.6	0.7	1.9 *	0.6	2.8	0.2435	1.8	0.7	1.7	0.7	1.9 *	0.7	1.6	0.4369
4. Acceptance	1.8	0.6	1.8	0.6	1.8 *	0.5	0.5	0.5823	2.0 *	0.5	1.8	0.6	−0.9	0.3271	2.0	0.6	1.8	0.6	1.8	0.6	1.8	0.4004	1.9	0.6	1.8 *	0.6	1.8	0.6	0.4	0.8083
5. Sense of humour	1.0	0.7	0.9	0.7	1.3	0.6	−1.9	0.0500	0.9	0.5	1.0	0.8	−0.1	0.8966	0.9	0.6	1.2	0.8	0.9	0.8	2.4	0.2948	1.0	0.7	1.2	0.7	0.7	0.7	4.4	0.1083
6. Religion	1.1	1.0	1.1	1.0	1.2	0.7	−0.7	0.4715	1.1	1.0	1.1	1.0	−0.1	0.9362	0.9	0.1	1.3	1.0	1.1	1.0	1.0	0.6044	1.0	1.0	1.6	1.0	0.9	0.9	3.6	0.1616
7. Use of emotional support	2.0 *	0.7	2.1 *	0.7	1.5	0.6	2.7	0.0065 **	1.8	0.7	2.0 *	0.8	1.0	0.2891	2.2 *	0.6	2.0 *	0.7	1.8	0.8	4.4	0.1111	2.1 *	0.6	1.9 *	0.8	1.8	0.9	2.3	0.3184
8. Use of instrumental support	1.8	0.6	1.9	0.6	1.3	0.5	2.9	0.0029 **	1.6	0.7	1.8	0.5	1.7	0.0910	1.9	0.5	1.8	0.7	1.7	0.5	1.9	0.3887	1.9	0.6	1.7	0.8	1.6	0.5	2.2	0.3322
9. Self-distraction	1.6	0.7	1.8	0.7	1.4	0.7	2.7	0.0067 **	1.6	0.7	1.7	0.8	0.3	0.7795	1.9	0.6	1.4	0.7	1.7	0.8	4.3	0.1180	1.7	0.6	1.4	1.0	1.7	0.8	0.8	0.6654
10. Denial	0.8	0.7	0.9	0.7	0.7	0.8	0.8	0.3735	0.8	0.7	0.8	0.7	0.2	0.8572	0.7	0.6	1.0	0.8	0.8	0.7	1.6	0.4465	0.7	0.7	1.2	0.7	0.8	0.7	4.8	0.0902
11. Venting	1.5	0.6	1.5	0.7	1.3	0.4	1.2	0.2420	1.4	0.6	1.5	0.6	0.4	0.7039	1.8	0.5	1.6	0.6	1.2	0.5	11.0	0.0041 **	1.7	0.6	1.5	0.6	1.2	0.6	9.3	0.0096 **
12. Substance use	0.5	0.8	0.4	0.7	1.1	1.1	−1.8	0.0657	0.8	0.9	0.4	0.8	−2.0	0.0426 **	0.4	0.7	0.8	1.1	0.4	0.6	0.6	0.7449	0.6	1.0	0.6	0.9	0.3	0.5	1.4	0.4931
13. Behavioural disengagement	0.8	0.6	0.8	0.7	0.6	0.4	0.4	0.7114	0.9	0.8	0.7	0.6	−1.2	0.2301	0.7	0.8	0.9	0.8	0.8	0.5	1.7	0.4325	0.7	0.8	0.9	0.4	0.8	0.6	1.6	0.4381
14. Self-blame	1.0	0.6	1.0	0.7	1.1	0.5	−0.6	0.5653	1.2	0.7	1.0	0.6	−1.1	0.2543	0.9	0.6	1.3	0.9	1.0	0.5	2.1	0.3533	1.1	0.8	1.1	0.6	0.9	0.6	0.3	0.8423

* The most commonly employed strategies; ** statistically significant values *p* < 0.05.

**Table 6:** The Spearman correlations between selected pairs of variables.

Pairs of Variables	rho	*p*
SWAQ and PSS-10	0.46	<0.0001 **
SWAQ and OLBI—exhaustion	0.39	0.0006 **
OLBI—exhaustion and Feeling of psychological strain related to work complexity	0.47	<0.0001 **
OLBI—exhaustion and Lack of rewards at work	0.41	0.0004 **
OLBI—exhaustion and Feeling of uncertainty resulting from workplace organisation	0.44	0.0001 **
OLBI—exhaustion and Social interactions	0.29	0.0151 **
OLBI—exhaustion and Feeling of threat	0.19	0.1030
OLBI—exhaustion and Physical inconvenience	0.21	0.0814
OLBI—exhaustion and Unpleasant work conditions	0.21	0.0758
OLBI—exhaustion and Lack of control	0.19	0.0992
OLBI—exhaustion and Lack of support	0.35	0.0025 **
OLBI—exhaustion and Sense of responsibility	0.18	0.1400
SWAQ and OLBI—disengagement	0.37	0.0017 **
OLBI—disengagement and Feeling of psychological strain related to work complexity	0.23	0.0510
OLBI—disengagement and Lack of rewards at work	0.45	<0.0001 **
OLBI—disengagement and Feeling of uncertainty resulting from workplace organisation	0.42	0.0003 **
OLBI—disengagement and Social interactions	0.16	0.1797
OLBI—disengagement and Feeling of threat	0.23	0.0499 **
OLBI—disengagement and Physical inconvenience	0.35	0.0023 **
OLBI—disengagement and Unpleasant work conditions	0.25	0.0343 **
OLBI—disengagement and Lack of control	0.35	0.0022 **
OLBI—disengagement and Lack of support	0.23	0.0554
OLBI—disengagement and Sense of responsibility	0.17	0.1686
PSS-10 and OLBI—exhaustion	0.41	0.0004 **
PSS-10 and OLBI—disengagement	0.10	0.3929

SWAQ, Subjective Work Assessment Questionnaire; PSS-10, Perceived Stress Scale; OLBI, Oldenburg Burnout Inventory; ** statistically significant values *p* < 0.05.

## Data Availability

All data are available from the corresponding author (dorota.wojtowicz@awf.wroc.pl).

## References

[1] Maslach C., Sęk H. (2010). Wypalenie zawodowe w perspektywie wielowymiarowej. Wypalenie zawodowe–przyczyny i zapobieganie.

[2] Pustułka-Piwnik U., Ryn Z.J., Krzywoszański Ł., Stożek J. (2014). Burnout syndrome in physical therapists–demographic and organizational factors. Med. Pr..

[3] Rotenstein L.S., Torre M., Ramos M.A., Rosales R.C., Guille C., Sen S., Mata D.A. (2018). Prevalence of Burnout Among Physicians: A Systematic Review. JAMA.

[4] Kowalczuk K., Zdańska A., Krajewska-Kułak E., Łukaszuk C., Van Damme-Ostapowicz K., Klimaszewska K., Kondzior D., Kowalewska B., Rozwadowska E. (2011). Stres w pracy pielęgniarek jako czynnik ryzyka wypalenia zawodowego. Probl. Piel..

[5] Friganović A., Selič P., Ilić B., Sedić B. (2019). Stress and burnout syndrome and their associations with coping and job satisfaction in critical care nurses: A literature review. Psychiatr. Danub..

[6] Głębocka A., Wilczek-Rużyczka E. (2016). Zachowania dehumanizujące pacjentów z perspektywy pracowników medycznych w oparciu o skalę behawioralnych wskaźników. Psych. J..

[7] Rasmus P., Marcinkowska W., Cieleban N., Lipert A. (2020). Obciążenie pracą i radzenie sobie ze stresem a stan zdrowia pracowników systemu państwowego ratownictwa medycznego w kontekście work-life-balance. Med. Pr..

[8] Kowalska J., Wójtowicz D., Szczepańska-Gieracha J. (2021). Physical Activity and the Emotional State of Physiotherapy Students Who Finish Their Education. Int. J. Environ. Res. Public Health.

[9] Kowalska J., Pawik M., Wójtowicz D., Szczepańska-Gieracha J. (2020). Evaluation of mood, stress levels and sense of coherence in future physiotherapists. Work.

[10] Mikołajewska E. (2014). Stres związany z pracą i wypalenie zawodowe u fizjoterapeutów–przegląd literatury. Med. Pr..

[11] Kowalska J. (2011). Wypalenie zawodowe wśród polskich fizjoterapeutów. Postepy Rehabil..

[12] Bruschini M., Carli A., Burla F. (2018). Burnout and work-related stress in Italian rehabilitation professionals: A comparison of physiotherapists, speech therapists and occupational therapists. Work.

[13] de Araújo Silva T.L., Alchieri J.C. (2014). Socioeconomic and demographic aspects related to stress and the burnout syndrome among Brazilian physiotherapists. Salud Mental..

[14] Carmona-Barrientos I., Gala-León F.J., Lupiani-Giménez M., Cruz-Barrientos A., Lucena-Anton D., Moral-Munoz J.A. (2020). Occupational stress and burnout among physiotherapists: A cross-sectional survey in Cadiz (Spain). Hum. Resour. Health.

[15] Lee S.-J., Jung S.I., Kim M.-G., Park E., Kim A.-R., Kim C.H., Hwang J.-M., Jung T.-D. (2021). The Influencing Factors of Gender Differences on Mental Burdens in Young Physiotherapists and Occupational Therapist. Int. J. Environ. Res. Public Health.

[16] Carpi M., Bruschini M., Burla F. (2021). HSE Management Standards and burnout dimensions among rehabilitation professionals. Occup. Med..

[17] Durand A.-C., Bompard C., Sportiello J., Michelet P., Gentile S. (2019). Stress and burnout among professionals working in the emergency department in a French university hospital: Prevalence and associated factors. Work.

[18] Baudry M., Briansoulet M., Perrochon A. (2020). The relationship between burnout and the mode of practice of physiotherapists. Kinesitherapie.

[19] Deneva T., Ianakiev Y., Keskinova D. (2019). Burnout Syndrome in Physicians—Psychological Assessment and Biomarker Research. Medicina.

[20] Ogińska-Bulik N., Juczyński Z. (2009). Narzędzia Pomiaru Stresu i Radzenia Sobie ze Stresem.

[21] Humeniuk E., Dąbska O., Pawlikowska-Łagód K. (2016). Stres zawodowy fizjoterapeutów–badania w wybranych oddziałach szpialnych. J. Educ. Health Sport.

[22] Dudek B., Waszkowska M., Merecz D., Hanke W. (1999). Ochrona Zdrowia Pracowników Przed Skutkami Stresu Zawodowego.

[23] Boland L.L., Kinzy T.G., Myers R.N., Fernstrom K.M., Kamrud J.W., Mink P.J., Stevens A.C. (2018). Burnout and exposure to critical incidents in a cohort of emergency medical services workers from Minnesota. West J. Emerg. Med..

[24] Naczelna Izba Lekarska [Internet] Deklaracja Helsińska Światowego Stowarzyszenia Lekarzy (WMA) Etyczne zasady prowadzenia badań medycznych z udziałem ludzi Przyjęta przez 18 Zgromadzenie Ogólne Światowego Stowarzyszenia Lekarzy (WMA), Helsinki, Finlandia, czerwiec 1964 r. i zmieniona przez: 64 Zgromadzenie Ogólne WMA, Fortaleza, Brazylia, październik 2013 r.; [cited 2021 Apr 22]. https://nil.org.pl/uploaded_files/art_1585807090_deklaracja-helsinska-przyjeta-na-64-zo-wma-pazdziernik-2013-pelny-tekst.pdf.

[25] Demerouti E., Bakker A., Vardakou I., Kantas A. (2003). The convergent validity of two burnout instruments: A multitrait-multimethod analysis. Eur. J. Psychol. Assess..

[26] Chirkowska-Smolak T. (2018). Polska adaptacja kwestionariusza do pomiaru wypalenia zawodowego OLBI. Studia Oeconomica Posnaniensia.

[27] Kozak S. (2009). Patologie w Środowisku Pracy: Zapobieganie i Leczenie.

[28] Sęk H. (2010). Uwarunkowania i mechanizmy wypalenia zawodowego w modelu społecznej psychologii poznawczej. Wypalenie Zawodowe–Przyczyny i Zapobieganie.

[29] Sochocka L., Wojtyłko A., Grad I., Kiliś-Pstrusińska K. (2012). Spostrzeganie stresu zawodowego przez pracowników ochrony zdrowia. Fam. Med. Prim. Care Rev..

[30] Adamus M.M., Owczarek K., Białoszewski D., Białoszewski D., Wroński Z. (2017). Wybrane aspekty psychologiczne w zawodzie fizjoterapeuty. Fizjoterapeuta w Polsce.

[31] Bejer A., Domka-Jopek E., Probachta M., Lenart-Domka E., Wojnar J. (2019). Burnout syndrome in physiotherapists working in the Podkarpackie province in Poland. Work.

[32] Wrzesińska M., Rasmus P., Wicherska K., Krukowska J. (2015). Wypalenie zawodowe a zmienne demograficzne i psychospołeczne u aktywnych zawodowo fizjoterapeutów. Zdrowie Publiczne i Zarządzanie.

[33] Pniak B., Leszczak J., Adamczyk M., Rusek W., Matłosz P., Guzik A. (2021). Occupational burnout among active physiotherapists working in clinical hospitals during the COVID-19 pandemic in south-eastern Poland. Work.

[34] Stanton A.L., Snider P.R. (1993). Coping with a breast cancer diagnosis: A prospective study. Health Psychol..

[35] Li L., Ai H., Gao L., Zhou H., Liu X., Zhang Z., Sun T., Fan L. (2017). Moderating effects of coping on work stress and job performance for nurses in tertiary hospitals: A cross-sectional survey in China. BMC Health Serv. Res..

[36] Paszkowska M. (2018). Wykonywanie zawodu fizjoterapeuty w uzdrowisku. Praktyczna Fizjoterapia i Rehabilitacja.

[37] Jaracz K., Górna K., Konieczna J. (2005). Burnout, stress and styles of coping among hospital nurses. Roczniki Akademii Medycznej w Białymstoku.

[38] Kawa R., Pisula E., Tomaszewski P. (2009). Professional burnout in therapists working with children with autism. New Ideas in Studying and Supporting the Development of Exceptional People.

[39] Baka Ł., Basińska B. (2016). Psychometryczne właściwości polskiej wersji Oldenburskiego Kwestionariusza Wypalenia Zawodowego (OLBI). Med. Pr..

